# Correlation between miRNA-124, miRNA-544a, and TNF-α levels in acute spinal cord injury

**DOI:** 10.1038/s41393-022-00763-4

**Published:** 2022-03-16

**Authors:** Xiaomin Ma, Tao Ma, Long Chang, Xiaolei Chen, Gen Xia, Chen Li, Huan Liu

**Affiliations:** 1 Orthopedics, General Hospital of Ningxia Medical University, Yinchuan, China; 2 Aab Cardiovascular Research Institute, Department of Medicine, University of Rochester School of Medicine and Dentistry, Rochester, NY USA

**Keywords:** Diagnostic markers, Acute inflammation

## Abstract

**Study design:**

Retrospective.

**Objectives:**

Acute spinal cord injury (ASCI) is caused by direct or indirect strikes from external forces on the spinal cord. Here, we investigated the correlation between the miR-124, miR-544a, and TNF-α levels in patients with ASCI, aiming to evaluate the potential usage of miR-124 and miR-544a in ASCI diagnosis.

**Setting:**

University/hospital.

**Methods:**

A total of 90 (58 male/32 female) ASIA patients and 15 (9 male/6 female) control patients (with acute limb trauma) were involved in the presented study. The ASIA patients were further subclustered based on the International Standards for the Neurological Classification of SCI (ISNCSCI) exam. 30 (18 male/12 female)cases were determined to have complete spinal cord injury (CSCI) and classified as ASIA grade A (Complete); 30 (20 male/10 female) cases were determined to have incomplete spinal cord injury (ISCI) and classified as ASIA grade B (sensory incomplete), C (motor incomplete), or D (motor incomplete); 30 (20 male/10 female) cases were determined to have normal neurological function (NNF) and classified as ASIA grade E (Normal). Plasma miR-124, miRNA-544a, and tumor necrosis factor-alpha (TNF-α) levels were measured from the blood samples collected 24 h, 48 h, and 72 h after trauma.

**Results:**

The levels of miR-124 and miR-544a in the CSCI and ISCI groups were significantly higher than those of the NNF and the control group 24 h after injury (*P* < 0.05). The increased levels gradually declined from 24 h to 72 h after injury. The area under the receiver operating characteristic curve (ROC) of miR-124, miR-544a and TNF-α 24 h after trauma in patients with acute spinal cord injury were 0.948 [95% CI (0.890, 1.000)], 0.815 [95% CI (0.638, 0.994)] and 0.770 [95% CI (0.641, 0.879)], respectively.

**Conclusion:**

The miRNA-124 and miRNA-544a levels increased significantly in ASCI patients compared with control patients 24 h after injury. These increased levels gradually reduced from 24 h to 72 h after injury. There is a strong positive correlation between miRNA-124, miRNA-544a, and acute spinal cord injury.

**Sponsorship:**

The present study was supported by a University-level project of Ningxia Medical University (Project Number: XY2017147).

## Introduction

Caused by direct or indirect strikes from external forces, acute spinal cord injury (ASCI) ASCI has a poor prognosis with high disability and mortality [1], resulting in lifelong physical dysfunction in most patients. Despite improvements in medical care and health care services, the incidence of ASCI has been increasing [2]. A scientific report shows that there are around 11,000 new cases of ASCI each year in the United States [3]. The increasing incidence of ASCI is probably due to increasing traffic accidents and fall injuries [2], causing great pain and burden to patients and their families [4]. Even though significant efforts have been taken to prevent the destructive effect of ASCl on the patients’ life quality, the physiopathology of spinal cord injury and self-regeneration remain unclear. Some published studies showed that the spinal cord does not have self-regeneration ability after injury [5]. Nevertheless, other studies revealed that neuroregeneration could promote ASCI recovery [6]. Despite the controversy, inflammation caused by cytokines and other small molecules has been agreed as the most common pathological process of ASCI [7]. However, the dynamic and complex production of cytokines and other small molecules after ASCI remains unclear. Thus, Deepening our understanding of this process is in demand to improve our knowledge about ASCI and identify potential therapeutic targets.

Previous studies have shown that post-transcriptional regulation of genes plays a central role in inflammation and the development of ASCI [8, 9]. It is well known that microRNA (miR) plays a vital role in post-transcriptional regulation, and the function of miR has gained significant interest in recent years [10, 11]. miR is an endogenously expressed single-stranded non-coding small RNA composed of 21–25 nucleotides. In humans, miRs bind to the 3’UTR of the target mRNA to inhibit the translation process or promote mRNA degradation to regulate gene expression at the post-transcriptional level [12, 13]. miRs have vast implications in many central nervous system diseases [14]. Several studies indicate that miRs contribute to the pathogenesis of SCI [15–17]. Animal studies have confirmed that serum levels of miR-124 and miR-544a change in the injured central nervous system [10, 15–17], simultaneously as other inflammatory factors, including tumor necrosis factor-α (TNF-α), which has been shown related to spinal cord injury [18–20]. Although several publicans have reported the roles of miRs in SCI, whether miRs can be used as biomarkers to predict ASCI prognosis remains unclear. Our study explores the diagnostic value of miR-124 and miR-544a for ASCI caused by acute spinal trauma, aiming to increase our knowledge about the physiopathology of ASCI and provide insight into ASCI diagnosis.

## Materials and methods

Retrospectively, this study was conducted on patients with acute spinal trauma admitted to the emergency department at the General Hospital of Ningxia Medical University from February 2018 to December 2020. A total of 90 (58 male/32 female) ASCI cases and 15 (9 male/6 female) control cases (patients with acute limb trauma hospitalized during the same period), with a mean age of 44.04 years, were involved. The 90 ASCI cases were further classified as complete spinal cord injury (CSCI), incomplete spinal cord injury (ISCI), or normal neurological function (NNF) according to the American Spinal Injury Association (ASIA classification) impairment scale (AIS) [21] determined by the International Standards for the Neurological Classification of SCI (ISNCSCI) exam. Thirty (18 male/12 female) cases were classified as CSCI and assigned as ASIA grade A (Complete); thirty (20 male/10 female) cases were classified as ISCI, including 13 (6 male/7 female) ASIA grade B (sensory incomplete) cases, 9 (6 male/3 female) ASIA grade C (motor incomplete) cases, and 8 (4 male/4 female) ASIA grade D (motor incomplete) cases; thirty (20 male/10 female) cases were classified as NNF and assigned as ASIA grade E (Normal). Clinical data of the involved cases were collected following approved protocols of the Committee of the General Hospital of Ningxia Medical University with written informed agreements obtained from patients.

Inclusion criteria: (1) ASCI patients meet the relevant diagnostic criteria in the acute spinal cord injury management guidelines issued by the 2013 American Congress of Neurosurgeons (CNS) and American Association of Neurosurgeons (AANS); Patients with acute limb trauma have clear images scientific diagnosis. (2) Patients were admitted to the hospital within 24 h from injury. (3) Patients are ≥ 18 years old.

Exclusion criteria: (1) Patients with a history of the spinal cord or craniocerebral trauma and surgery within half a year. (2) Patients with a large area of myocardial infarction or acute coronary syndrome, severe arrhythmia, acute respiratory distress syndrome, or other severe cardiopulmonary diseases. (3) Acute severe head injury (severe brain trauma, massive cerebral hemorrhage, extensive cerebral infarction, severe brain stem injury, etc.). (4) Pregnant women. (5) Patients with a history of acute SCI or head injury. (6) Patients with malignant tumors, acute and chronic infections, severe liver or kidney dysfunction, cerebrovascular diseases, autoimmune diseases, and blood system disease.

### Measurement of serum miR-124, miR-544a, and TNF-α

#### Reagents and equipment

TNF-α radioimmunoassay kit: Catalog # BY20190158, Interassay CV: 7.5%, Intraassay CV: 4.4%, Beijing Beiya Institute of Immunobiological Technology. RNA extraction reagent: TRI Reagent® BD (TB 126), Catalog # TB126, Interassay CV: < 10%, Intraassay CV: < 10%, MRCGENE. Real-time catastrophe quantitative PCR kit: One-Step TB Green PrimeScript RT-PCR Kit II (Perfect Real Time), Catalog # RR086B, Interassay CV: < 10%, Intraassay CV: < 10%, takarabio. Reverse transcription kit: Efficient preparation of cDNA: PrimeScript Reverse Transcriptase, Catalog # 2680B, Interassay CV: < 10%, Intraassay CV: < 10%, takarabio. Wizard2 2-Detector Gamma Counter, 550 samples, Catalog # C 2470–0020, PerkinElmer, Germany. PCR instrument: ABI Geneamp 9700 PCR - Thermal Cycler, Catalog # ABI-97, ABI, USA. NanoDrop™ 2000/2000c Spectrophotometers, Catalog # ND2000CLAPTOP, Thermo Fisher Scientific, USA.

#### Specimen collection

Approximately 4 ml of peripheral venous blood was collected, placed in an EDTA-K2 anticoagulant tube, and stored at 4 °C after the patient was admitted to the hospital (within 24 h after the trauma), at 48 h, and 72 h after trauma.

#### Determination of plasma miR-124 and miR-544a level

Real-time fluorescent quantitative PCR technology (RT-qPCR) was used to determine plasma miR-124 and miR-544a levels. Peripheral venous blood samples were centrifuged at 3000 g/10 min to collect plasma, which was then transferred to new 1.5 ml EP tubes and centrifuged at 12000 g/10 min. Total RNA was extracted from 200 μl of aliquoted plasma using RNA extraction reagent. ND2000C UV spectrophotometer was used to determine the concentration and purity of the RNA. RNA samples with A260/A280 between 1.8 and 2.1 were used for cDNA synthesis by reverse transcription kit. For miRs measurements using RT-qPCR, the primer design, and synthesis was completed by Guangzhou Ruibo Biotechnology Co., Ltd. miR-124: Forward: TTC ACA GCG GAC CTT GA, Reverse: GAA CAT GTC TGC GTA TCT C; miR-544a: Forward CAG ATT CTG ATT CAG GGA C CA AG; Reverse: CCA CAG ACC GGC GGT ATT A; U6 Forward: GCT TCG GCA GCA CAT ATA CTA AAA T, Reverse: CGC TTC ACG AAT TTG CGT GTC AT. RT-qPCR was performed as follows: 95 °C pre-denaturation for 30 s; 95 °C denaturation for 3 s, 60 °C annealing and extension for 30 s, 40 cycles; 95 °C 15 s, 60 °C 1 min, 95 °C 15 s, 60 °C 15 s. Melting curves were drawn, and plasma miR-124 and miR-544a levels were calculated using the 2-ΔΔCt method with the internal control reference gene U6.

#### Determination of serum TNF-α level

Immunoassay was used to determine serum TNF-α level strictly following the instructions provided in the kit.

#### Statistical analysis

SPSS 21.0 statistical software was used for data analysis. Continuous variables were tested for normality. Measurement data conforming to the normal distribution were expressed as ($\bar \chi$ ± *s*). A single-factor analysis of variance performed the comparison between multiple groups. SNK-q test was used for pairwise comparison; *χ*^2^ test was used for count data comparison; Predict Receiver operating characteristic (ROC) model curve was used to evaluate the clinical significance of miR-124, miR-544a, and TNF-α in the diagnosis of early infection. *P* < 0.05 was considered to show a statistically significant difference.

## Results

### Characteristics of the study population

According to the American Spinal Injury Association (ASIA classification) impairment scale (AIS), the presented study involved 30 (18 male/12 female) ASIA grade A injuries (33.3%) cases, 13 (6 male/7 female) ASIA grade B injuries (14.4%) cases, 9 (6 male/3 female) ASIA grade C injuries (10%) cases, 8 (4 male/4 female) ASIA grade D injuries (8.9%) cases, and 30 (20 male/10 female) ASIA grade E injuries (33.4%) cases. We first summarized age, gender, injury severity scores, length of stay, and etiology of injury of the involved cases. As shown in Table 1, there was no statistically significant difference in age, gender, and etiology of injury among CSCI, ISCI, and NNF groups (*P* > 0.05), However, injury severity scores and length of stay among CSCI, ISCI, and NNF groups were significantly different. (*P* < 0.05).

**Table 1:** Characteristics of CP, CSCI, ISCI, and NNF.

Group	CP (*n* = 15)	CSCI (*n* = 30)	ISCI (*n* = 30)	NNF (*n* = 30)	*F* (*χ*^2^)	*P*
Age(years)	44.3 ± 11.2	45.5 ± 9.8	43.5 ± 10.8	46.1 ± 10.5	0.33	0.83
Sex					0.34	1.25
male	9 (60%)	18 (60%)	20 (66.7%)	20 (66.7%)		
female	6 (40%)	12 (40%)	10 (33.3%)	10 (33.3%)		
ISS		32.1 ± 15.3	24.8 ± 9.1	18.4 ± 8.9	8.26	0.02
Length of stay		47.3 ± 18.6	32.6 ± 11.3	25.8 ± 10.2	7.35	0.48
Etiology of injury					1.389	0.226
Traffic accident (%)		17 (57%)	14 (47%)	21 (70%)		
Fall (%)		6 (20%)	8 (27%)	4 (13%)		
Object hit (%)		5 (17%)	7 (23%)	4 (13%)		
Sports (%)		2 (6%)	1 (3%)	1 (3%)		

^a^χ^2^ test; Data are presented as mean ± standard deviation, *CSCI* Complete spinal cord injury, *ISCI* Incomplete spinal cord injury, *NNF* Normal neurological function, *CP* Control group, *ISS* Injury Severity Scores, *F* F-test results; *P* P-value of the F-test. *P* < 0.05 was considered to show a statistically significant difference.

### Comparison of the serum levels of miR-124, miR-544a, and TNF-α in each group in ASCI

We next examined the changes in serum levels of miR-124, miR-544a, and TNF-α in each ASCI group. As shown in Table 2, there was a statistically significant difference in the serum levels of miR-124, miR-544a, and TNF-α among three groups at 24 h after trauma (*P* < 0.05). The levels of miR-124, miR-544a, and TNF-α in the CSCI group and the ISCI group were significantly higher than those of the NNF group and the control group 24 h after injury (*P* < 0.05).

**Table 2:** miR-124, miR-544a, and TNF-α levels in CP, CSCI, ISCI, and NNF groups.

Group	*n*	miR-124	miR-544a	TNF-α (µg/L)
CP	15	0.98 ± 0.12	0.97 ± 0.23	1.65 ± 0.17
CSCI	30	3.51 ± 0.27	4.57 ± 0.58	2.02 ± 0.25
ISCI	30	2.38 ± 0.35	3.26 ± 0.23	1.83 ± 0.41
NNF	30	1.25 ± 0.19	1.55 ± 0.29	1.75 ± 0.19
F		28.38	25.61	3.69
P		0.01	0.01	0.025

Serum miR-124, miR-544a, and TNF-α levels of each group at 24 h after injury are presented as mean ± standard deviation, *CSCI* The complete spinal cord injury group, *ISCI* The incomplete spinal cord injury group, *NNF* The normal neurological function, *CP* Control group, *F* F-test results, *P* P-value of the F-test. *P* < 0.05 was considered to show a statistically significant difference.

### Comparison of the serum levels of miR-124, miR-544a, and TNF-α in the same group at 24, 48, and 72 h

**Fig. 1:**
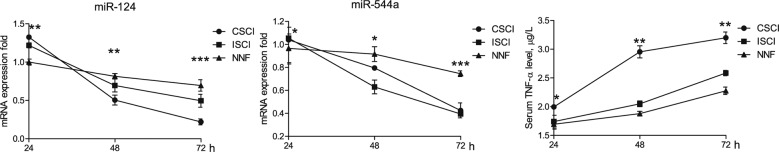
Levels of miR-124, miR-544a, and TNF-α in each group at 24, 48, and 72 h. CSCI The complete spinal cord injury group, ISCI The incomplete spinal cord injury group, NNF The normal neurological function; h Hours, *P* P-value of the F-test. ^*^P < 0.05;^**^P < 0.1; ^***^P < 0.001*. P* < 0.05 was considered to show a statistically significant difference.

We then examined the changes in serum levels of miR-124, miR-544a, and TNF-α with time. As shown in Fig. 1, there was a statistically significant difference in the serum levels of miR-124, miR-544a, and TNF-α in each group at 24, 48, and 72 h time points (*P* < 0.05). In each group, the levels of miR-124 and miR-544a decreased from 24 h to 72 h after injury, while the levels of TNF-α continuously increased over time (*P* < 0.05).

### Receiver operating characteristics (ROC) of miR-124, miR-544a, and TNF-α

**Fig. 2:**
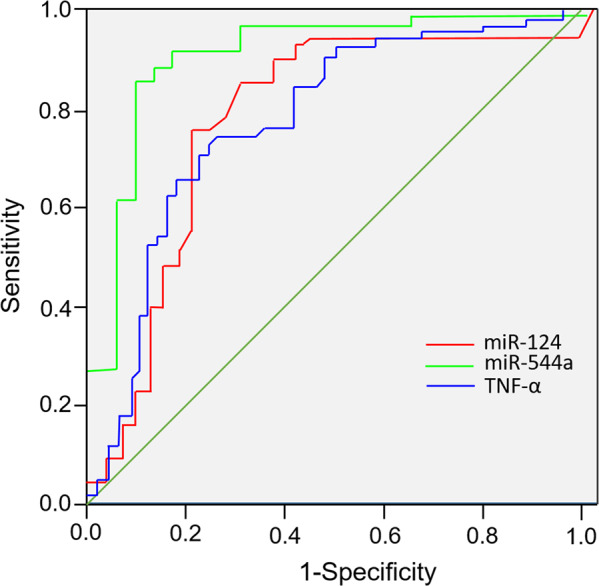
Receiver operating characteristic (ROC) curve analysis of the variations of miR-124, miR-544a, and TNF-α in patient with ASCI at 24 h. *P* < 0.05 was considered statistically significant.

We applied the Receiver operating characteristics (ROC) method to evaluate the diagnostic values of peripheral venous blood miR-124, miR-544a, and TNF-α levels in ASCI. As shown in Fig. 2, miR-124, miR-544a, and TNF-α all demonstrated good sensitivity and specificity in diagnosis of ASCI when using at the optimal thresholds. (miR-124, area under curve (AUC): 0.948 [95% CI (0.890, 1.000)], threshold: 2.27 µg/L; miR-544a, AUC: 0.815 [95% CI (0.792, 0.823)], threshold: 3.24 µg/L; TNF-α, AUC: 0.770 [95% CI (0.641, 0.879), threshold: 1.82 µg/L.].

## Discussion

In the presented study, we measured serum levels of miR-124, miR-544a, and TNF-α in the peripheral venous blood of 90 ASCI patients and 15 control patients. We examined the correlation between them and acute spinal cord injury and evaluated the potential to use miR-124 and miR-544a as diagnostic markers for ASCI. Our results showed that serum levels of miR-124 and miR-544a increased in spinal cord injury groups compared with normal groups 24 h after injury. These increased levels gradually reduced from 24 h to 72 h after injury. Our results also suggested that miR-12, miR-544a, and TNF-α were potentially sensitive and specific diagnostic biomarkers for acute spinal cord injury.

Traditionally, ASCI diagnosis depends on thorough patient history, standardized neurological physical examinations, and radiographic imaging of the spinal cord [22]. These procedures require professional medical teams and equipment, often unavailable in some developing countries and areas lacking sophisticated medical resources [23]. Besides, patients often suffer from pain and post-traumatic mental disorder while performing these tests [24]. What is worse, the time-consuming tests could potentially cause doctors to miss crucial time to treat patients with ASCI [24]. Therefore, developing quick, reliable, and practical tools to diagnose the severity and predict the progression of ASCI is crucially important. Assessment of biomarker concentrations in peripheral blood is a quick, non-invasive and practical method to diagnose ASCI and assess the severity of SCI. Based on this idea, multiple potential ‘biomarkers’ have been measured and identified as significantly changed in ASCI patients. Most of these ‘biomarkers’ are inflammatory factors, including pro-inflammatory cytokines, such as TNF and interleukins, which is expected since increased inflammation has been well-evident in the spinal cord within minutes of injury [25, 26]. However, the sensitivity and specificity of these inflammatory factors are either not evaluated or not high enough to always accurately distinguish the severity of acute spinal cord injury. Our study, for the first time, evaluates the possibility of using miRs instead of inflammatory factors as diagnostic biomarkers of ASCI. Our receiver operating characteristics (ROC) results demonstrated the high sensitivity and specificity of miR-124, miR-544a as diagnostic markers of ASCI. Besides, our data showed that miR-124 and miR-544a are highly correlated with SCI severity and can be potentially used as sensitive indicators of SCI severity. In addition, We also suggested the optimal cut-off for diagnosis, which may be applied to potential clinical applications.

Previous studies have indicated that miR-124 plays a critical role in central nervous system diseases, including cerebral ischemia, epilepsy, and Parkinson’s disease [27]. Our results demonstrated increased serum levels of miR-124 in acute spinal cord injury and the highest serum levels of miR-124 in cases with complete spinal cord injury 24 h after injury. These are similar to previous studies showing miR-124 increases by secondary injury after ASCI in mice and other mammals [28, 29]. Besides, we found miR-124 level decreased during acute spinal cord injury development, which is in accord with the previous discovery showing that the low level of miR-124 is associated with worse recovery [30]. In addition, our ROC analysis shows that miR-124 has high sensitivity and specificity to diagnose acute spinal cord injury when used with the optimal cut-off of 2.27 µg/L, indicating clinic application of miR-124.

Unlike miR-124, miR-544a has never been linked with central nervous system diseases until our study. MiR-544a is a recently discovered miRNA located at 14q32 and has been shown to affect multiple metastasis-associated pathways by inhibiting target genes [17]. Recent studies have shown that miR-544a induces endothelial to mesenchymal transition in gastric tissue by activating the WNT signaling pathway [17]. Our studies, firstly, indicate the potential of using miR-544a as sensitive and specific diagnostic biomarkers to evaluate acute spinal cord injury. MiR-544a is shown to increase during acute spinal cord injury, which is correlated with its role in immune and inflammation responses [17]. Similar to miR-124, miR-544a also decreased from 24 h to 72 h after acute spinal cord injury and has high sensitivity and specificity to determine the severity of acute spinal cord injury. The optimal cut-off is 3.24 µg/L. Besides, serum levels of miR-124 and miR-544a may be used together to evaluate the severity of acute spinal cord injury with higher specificity.

## Limitations

Although our study demonstrated promising biomarkers, there are still some limitations in this study. Limitations include (a) This study only enrolls patients with ASCI from the Ningxia area, and thus it may not fully represent ASCI patients in the global trend. (b) Our study only focuses on two miRNAs, including miR-124 and miR544a. A comprehensive analysis using a high throughput technique to screen for the miRNAs profiles at several time points after the injury is needed to identify diagnostic markers. (c) Our study only measures miR-124 and miR544a in a small population, and future study is needed to determine the value of miR-124 and miR544a as diagnostic markers in a large population.

## Conclusion

In conclusion, our study demonstrated that miR-124 and miR-544a have the potential to be used as diagnostic markers for acute spinal cord injury with high sensitivity and specificity. These miRNAs can help quickly identify patients with acute SCI in the acute phase of trauma (within 24 h) and save time for surgery and treatment.

## Data Availability

The data that support the findings of this study are available from the corresponding author, Xiaomin Ma, upon reasonable request.
